# Electro-Mechanical Characterisation and Damage Monitoring by Acoustic Emission of 3D-Printed CB/PLA

**DOI:** 10.3390/ma17051047

**Published:** 2024-02-24

**Authors:** Laurane Roumy, Thuy-Quynh Truong-Hoang, Fabienne Touchard, Colin Robert, Francisca Martinez-Hergueta

**Affiliations:** 1 Département Physique et Mécanique des Matériaux, Institut Pprime, CNRS-ENSMA-Université de Poitiers, ENSMA, 1 Av. C. Ader, B.P. 40109, 86961 Futuroscope, France; 2 ESTACA’Lab-Laval, ESTACA, 53000 Laval, France; thuy-quynh.truong-hoang@estaca.fr; 3 Institute for Infrastructures and Environment, School of Engineering, University of Edinburgh, Edinburgh EH8 9YL, UK; francisca.mhergueta@ed.ac.uk; 4 Institute for Materials and Processes, School of Engineering, University of Edinburgh, Edinburgh EH8 9YL, UK; c.robert@sheffield.ac.uk

**Keywords:** additive manufacturing, 4D printing, tensile test, resistivity, craze, repeated progressive tensile test, SEM, smart material, shape memory polymer

## Abstract

Even though the influence of the printing direction on the mechanical properties of 3D-printed samples by fused filament fabrication is established in the literature, very little is known about mechanical and electrical coupling. In this study, electrically conductive polylactic acid filled with carbon black particles undergoes monotonic and repeated progressive tensile loading to better understand the influence of the printing direction on the electro-mechanical properties of three-dimensional-printed samples. The objective is to analyse the electro-mechanical behaviour of this composite for its potential application as an actuator. The classical laminate theory is also applied to evaluate the relevance of this theory in predicting the mechanical characteristics of this material. In addition, a comprehensive damage analysis is performed using acoustic emission, infrared thermography, scanning electron microscopy, and X-ray microcomputed tomography imaging. Results show that the degradation of the mechanical and electrical properties is highly influenced by the printing direction. The appearance and development of crazes in 0° filaments are highlighted and quantified. The conclusions drawn by this study underline the interest in using longitudinal and unidirectional printing directions to improve the conductive path within the samples. Furthermore, the evolution of the resistance throughout the experiments emphasizes the need to control the implemented voltage in the design of future electro-thermally triggered actuators.

## 1. Introduction

Polylactic acid (PLA) is a bio-sourced thermoplastic polymer used in numerous domains, such as food packaging or bio-degradable surgical stitches. In three-dimensional (3D) printing, it is the most widely used polymer because of its low melting point, which is between 190 °C and 220 °C, and its optimal consolidation once printed. In addition, 3D printing allows the optimisation and automation of production, leading to less waste and the possibility of using recycled thermoplastic materials [1] for a manufacturing process that is more respectful of the environment. Nowadays, the shape memory capability of PLA is of high interest for the manufacturing of four-dimensional (4D) printed components. This fabrication process consists of the use of shape memory polymers (SMPs) [2], or smart materials, for the 3D printing of components that, once subjected to an external stimulus, can move from a pre-programmed to a permanent shape, the fourth dimension referring to time [3,4,5]. PLA reacts to heat once filled with carbon black (CB) particles; CB/PLA can therefore be heated by the Joule effect as an electro-conductive SMP [6,7,8]. This property enables actuators’ motion without any motors or external action. It finds applications in medicine [9], self-healing structures [10], soft robotics [11,12,13], and aerospace [14].

Different kinds of 3D printing techniques exist [15,16,17]. In this study, fused filament fabrication (FFF) is used because of its affordability and ease of use. The printing process consists of the melting of a material through a heated nozzle to be deposited as filaments along a pre-programmed path to create a 3D structure [18]. However, from a scientific perspective, this additive manufacturing process is quite challenging to master, as the properties of the 3D-printed samples highly depend on the printing parameters [19,20,21]. As a consequence, the 3D-printed CB/PLA structures exhibit a more intricate electro-thermo-mechanical coupling, which is further challenging to control. In a previous study, our team analysed the electro-thermal behaviour of this material [22,23]. In terms of electro-mechanical coupling, to the knowledge of the author, only a few studies have partially considered the question for 3D-printed CB/PLA specimens. Tirado-Garcia et al. [24] studied the evolution of the electrical resistance during a tensile test of a 0° sample, whose filaments are printed along the longitudinal direction, which is the same as the loading direction. However, no other printing direction is considered. Crespo-Miguel et al. [25] studied different raster angles under tensile testing in environmental chambers to analyse the effect of various temperatures on the behaviour of the material as a thermo-mechanical coupling. In terms of electro-mechanical coupling, they realised tensile tests on their samples after being subjected to several voltages, but no monitoring of the resistance throughout the experiment was performed.

In this study, the mechanical and electrical coupling is pushed further by monitoring the evolution of the electrical resistance and calculating the inherent resistivity of 3D-printed CB/PLA samples subjected to uniaxial tensile loading. Four different configurations are studied: 0° samples with filaments deposited along the longitudinal length of the sample, parallel to the loading direction; 90° samples with filaments deposited in the transverse direction; ±45° samples with filaments deposited at ±45° compared to the longitudinal direction; and a quasi-isotropic (QI) stacking sequence with 0°, 90°, and ±45° layers. Furthermore, because CB/PLA is a non-linear elastoplastic material whose plastic behaviour is not well characterised yet, repeated progressive tensile tests were also carried out to measure the plastic response and its influence on resistivity.

In addition, the manufacturing process makes it possible to consider the classical laminate theory (CLT) as the resulting 3D specimens demonstrate anisotropic mechanical properties. Each printed filament can be assimilated into a continuous fibre. This analogy has already been studied for 3D-printed continuous carbon fibre composites [26], as well as short carbon fibre composites [27], and short wood fibre composites [28]. In this study, the CLT is applied to CB/PLA to verify the relevance of using it for such a composite manufactured by 3D printing.

To better understand the evolution of the mechanical properties of the 3D-printed CB/PLA under tensile loading, damage monitoring by acoustic emission was also conducted. The simultaneous use of acoustic emission and electrical measurements was reported in the literature on bonding joints for structural health monitoring [29]. Acoustic emission is a common technology used to assess the damage evolution within polymer matrix composites [30,31,32,33,34,35]. In additive manufacturing, this technique has also been applied to 3D-printed polymer composites filled with continuous fibres [36,37,38] or short flax fibres [39] as well as 3D-printed sandwich structures [40]. However, as far as the author knows, no acoustic emission on 3D-printed CB/PLA has been reported in the literature.

Thus, the aim of this study is to gain a better understanding of the mechanical and electrical coupling in the 3D-printed CB/PLA by performing multi-instrumented monotonic and repeated progressive tensile tests with the measurement of the electrical resistance as well as acoustic emission monitoring on unidirectional and multi-directional specimens. This study is completed by a CLT analysis to evaluate the mechanical behaviour of the material. To understand the failure mechanisms, images were taken by scanning electron microscopy (SEM) and microcomputed tomography (micro-CT) of the fracture surfaces, and tested samples were also realised.

## 2. Materials and Methods

### 2.1. CB/PLA and 3D-Printed Samples

In this study, the material considered is Proto Pasta’s electrically conductive composite PLA, which consists of polylactic acid filled with carbon black particles (CB/PLA). PLA is a shape memory polymer (SMP) commonly used in 3D printing, made conductive thanks to its 21.5% weight of CB [22]. Ultimaker printers (Utrecht, The Netherlands) with the Fused Filament Fabrication technology were used with a nozzle of 0.8 mm in diameter heated at 210 °C. The sample was 3D modelled thanks to the CAD software CATIA V5 to obtain a .stl file that was converted to a .gcode file thanks to Ultimaker Cura 4.11.0. This software was used to define the printing parameters and the path followed by the printer. The samples were printed with an infill density of 100% and a printing speed of 60 mm/s. The parameters of the filaments were set to a layer height of 0.1 mm and a line width of 0.7 mm and deposited on a bed heated at 60 °C. These parameters were optimised in a previous study [22].

**Figure 1:**
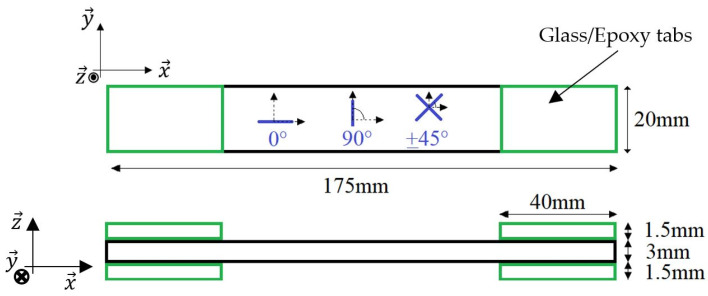
Dimensions of the rectangular tensile samples with the different raster angles.

Rectangular samples were manufactured following the standard test method for tensile properties of polymer matrix composite materials ASTM D 3039/3039M [41]. Their dimensions were 175 × 20 × 3 mm^3^ and rectangular glass/epoxy tabs were cut from a panel at a dimension of 40 × 20 × 1.5 mm^3^ (Figure 1). Three raster angles were studied: 0° filaments following the longitudinal length of the sample; 90° filaments that are perpendicular to this direction; and ±45° filaments that consist of an alternation of layers that are at ±45° compared to the longitudinal direction. Each sample consists of several layers with the same printing directions. A quasi-isotropic (QI) sample with a [0_6_/±45_5_/90_5_]_S_ stacking sequence was also manufactured.

**Figure 2:**
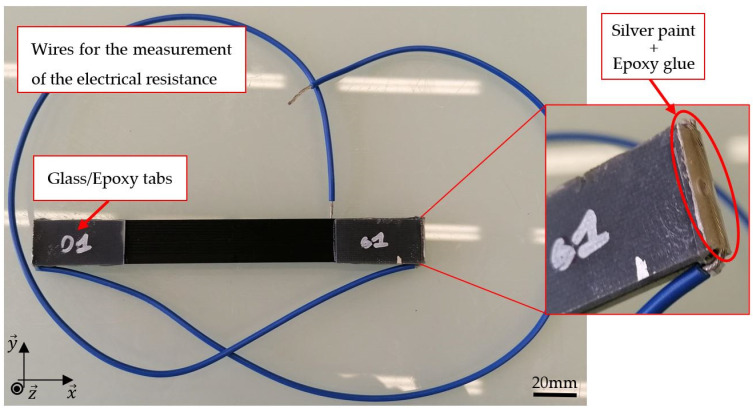
3D-printed CB/PLA tensile samples and focus on the glued wires for electrical measurements.

In order to measure the electrical resistance of the samples, Loctite MR 3863 silver paint from Henkel (Düsseldorf, Germany) was applied at the two extremities of their cross-section, and wires were glued with Araldite standard epoxy glue from VELCRO Brand (Manchester, NH, USA) (Figure 2).

### 2.2. Multi-Instrumented Tensile Tests

**Figure 3:**
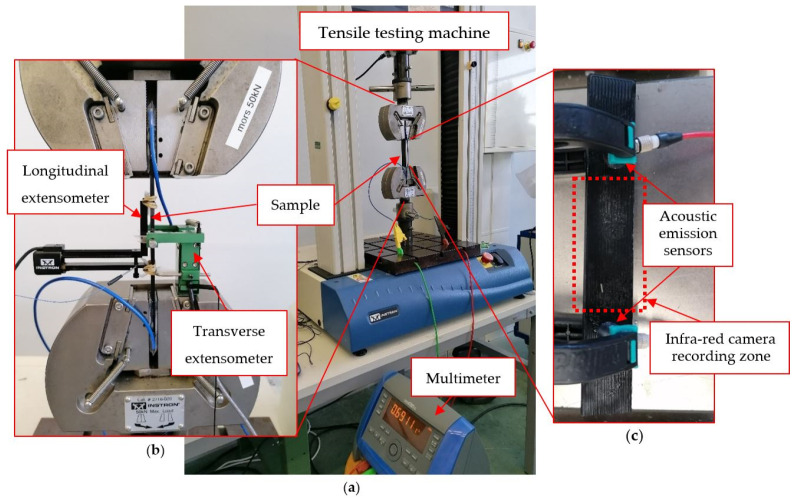
(**a**) Set-up for the measurement of the electrical resistance during tensile tests; (**b**) Focus on the sample and the measurement of the longitudinal and transverse strains; and (**c**) Acoustic emission sensors and thermal camera recording set-up.

Monotonic and repeated progressive tensile tests were conducted at a cross-head speed of 1 mm/min with the load applied along the $\vec{x}$ axis of the samples (Figure 1). The rectangular specimens were clamped in a 5982 INSTRON machine (Norwood, MA, USA) (Figure 3a) and instrumented with longitudinal and transverse extensometers to measure the corresponding strains (Figure 3b). A longitudinal gauge length of 50 mm was used. The sample was also connected to a 2-point probe MTX 3250 multimeter from Chauvin Arnoux (Asnières-sur-Seine, France) that measured the electrical resistance of the samples during the tests. An infrared A320 FLIR camera from TELEDYNE FLIR (Wilsonville, OR, USA) with a resolution of 320 × 240 pixels and a thermal sensitivity of less than 0.05 °C for temperatures higher than 30 °C was used to measure the temperature field at the surface of the samples during the tensile tests (Figure 3c). The hypothesis that the emissivity of the sample is equal to 1 is made, and the temperature of the environment is 20 °C. Seven to ten specimens were tested for each lay-up studied.

The electrical resistance *R*(*t*) measured during the tensile tests depends on the length *L*(*t*) and cross-section area *S*(*t*) of the sample at time *t*. To remove this dependency, the resistivity *ρ*(*t*), which is inherent to the material, was considered with the formula [42] (Equation (1)):

(1) $$\rho \left(t\right)=\frac{R\left(t\right)\cdot S(t)}{L(t)},$$

The hypothesis that the Poisson’s ratio *ν* is the same in directions *xy* and *xz* was made (Equation (2)):

(2) $$\nu_{xy}=\nu_{xz}.$$

Therefore, the transverse and out-of-plane strains, respectively, $\varepsilon_{yy}(t)$ and $\varepsilon_{zz}(t)$, were considered equal all along the experiment (Equation (3)):

(3) $$\varepsilon_{yy}(t)=\varepsilon_{zz}(t).$$

The section *S*(*t*) at any time could then be deduced (Equation (4)):

(4) $$S(t)=S_{0}{\left(1+\varepsilon_{yy}(t)\right)}^{2},$$

with *S*_0_ the initial cross-section of the sample. In addition, the evolution of the length of the sample *L*(*t*) depends on the length covered by the two tabs *L_T_*, the initial total length of the sample *L*_0_ and the longitudinal strain $\varepsilon_{xx}(t)$ (Equation (5)):

(5) $$L\left(t\right)=L_{T}+\left(L_{0}-L_{T}\right)\cdot (1+\varepsilon_{xx}\left(t\right)).$$

This way, the resistivity *ρ*(*t*) of the sample could be calculated throughout the tensile test (Equation (6)):

(6) $$\rho (t)=\frac{R(t)\cdot S_{0}{\left(1+\varepsilon_{yy}(t)\right)}^{2}}{L_{T}+\left(L_{0}-L_{T}\right)\cdot (1+\varepsilon_{xx}(t))},$$

with *R*(*t*) the measured electrical resistance, *L*_0_ and *S*_0_ the initial length and cross-section of the sample, respectively, *L_T_* the length of the two tabs, $\varepsilon_{xx}(t)$ and $\varepsilon_{yy}(t)$ the longitudinal and transverse strains measured by the extensometers, respectively. In addition to the measurement of the electrical resistance, acoustic emission was also performed. To the knowledge of the authors, no study dealing with these two means for 3D-printed materials was published in the literature.

Acoustic emission was used to obtain an in situ and non-destructive analysis of the damage process (Figure 3c). The transient elastic sound waves produced by the damage were recorded by two Euro Physical Acoustics piezoelectric sensors. The contact between the samples and the sensors was made with a silicone gel and maintained with plastic clamps. The acquisition system PCI-2 by Mistras Group (Sucy-en-Brie, France) worked with a preamplifier with a gain of 40 dB. The amplitude threshold was set to 35 dB. A maximum duration of 1000 μs, a PDT of 50 μs, an HDT of 100 μs, and an HLT of 300 μs were also the main parameters implemented. Before each test, a data acquisition calibration step was performed. It consisted of the measurement of the acoustic wave speed by breaking a pencil lead on the sample. In addition, events were determined by considering the hits that were measured by both sensors and that were localised within the gauge length of the sample.

**Figure 4:**
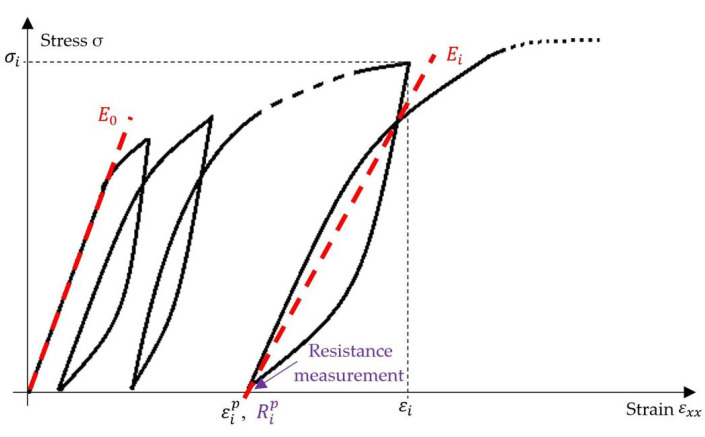
Schematic stress–strain loading-unloading curve of a repeated progressive tensile test and the definition of the main measurements.

Different parameters were deduced from the repeated progressive tensile tests (Figure 4). Five to six loading-unloading cycles were applied to each sample with an increment of a force of about 100 N and 200 N between two successive cycles. The Young’s modulus *E*_0_ was measured on the initial slope of the stress–strain curve during the first cycle. To analyse the evolution of the modulus throughout the tensile test, secant moduli were measured on the hysteresis loops obtained, as illustrated in Figure 4. For cycle i, the maximum stress $\sigma_{i}$ applied to the material and the permanent strain $\varepsilon_{i}^{p}$, corresponding to the strain once the sample was unloaded at the end of the cycle, were also measured. In addition, at the same time as the permanent strain, the permanent electrical resistance $R_{i}^{p}$ was measured, as the resistance of the sample after unloading. The last cycle was performed at up to 80% of the elongation at the break of the specimens.

### 2.3. Damage Observation

For microstructural observations, the samples were sputtered with a thin layer of gold before using a TESCAN VEGA3 scanning electron microscope (Brno, Czech Republic) with secondary electrons accelerated with a voltage of 5 to 10 kV. In addition, an UltraTom CT scanner manufactured by RX Solutions (Chavanod, France) was used with a voxel resolution of 12 μm. Its main component is a Hamamatsu microfocus sealed X-ray tube (Shizuoka, Japan) that operates within a range of 0 to 500 µA, 20 to 150 kV, for a maximum power of 75 W. The acquisitions presented here were performed at a current of 200 µA and a voltage of 50 kV.

## 3. Results and Discussion

### 3.1. Mechanical Properties and Classical Laminate Theory (CLT)

**Figure 5:**
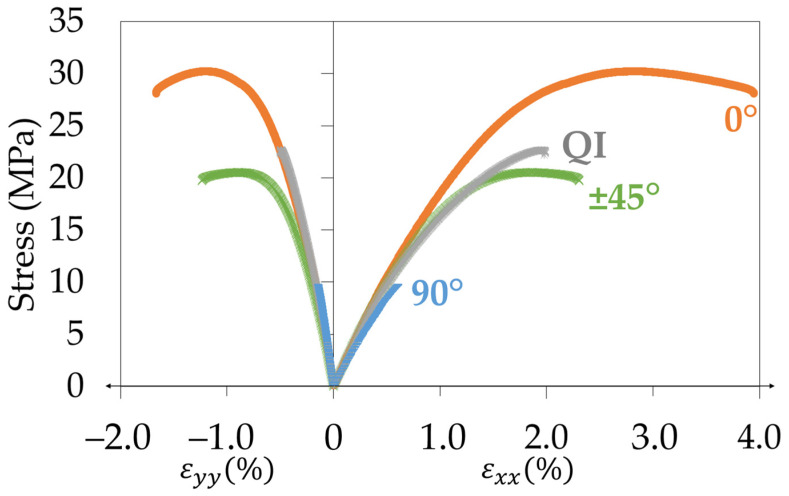
Representative stress–strain curves for the different raster angles and the QI sample.

A mechanical characterisation of 3D-printed CB/PLA samples was conducted. An example of the stress–strain curves obtained during monotonic tensile tests on the four different kinds of samples is presented in Figure 5, and the mechanical properties measured are summarised in Table 1. The highest Young’s modulus of 2542 MPa is obtained for the 0° sample, and the lowest at 1744 MPa is for the 90° sample. The QI sample has a stiffness of 2187 MPa, similar to the ±45° sample with a stiffness of 2108 MPa. In regards to the Poisson’s ratio, the values slightly decrease from 0.42 for a 0° sample, to 0.40 for a ±45° sample and 0.37 for a QI sample. The 90° samples have a fairly lower Poisson’s ratio equal to 0.25, which means that these kinds of samples are less compressible than the others. It is explained by the fact that the stiffness of the filaments acts against the decrease in their length in the transverse direction of the 90° specimens. In terms of maximum stress, failure stress, and elongation at break, the results show the poor mechanical properties of the 90° specimens in comparison to the other raster angles. It is explained by the weakness of the inter-filament interface that endures the stress increase in this structure, whereas for the other printing directions, the filaments take the stress over. The QI samples present a higher maximum stress and failure stress than the ±45° samples, but a lower elongation at break. It can be explained by two coupled mechanisms: the weakening of the structure by the 90° layers and the strengthening due to the 0° layers. Overall, the 0° specimens have much better mechanical properties than the other raster angles.

Thus, the 3D-printed CB/PLA has demonstrated a strong anisotropy, as the modulus of the 0° samples is 46% higher than that of the 90° samples. One may wonder if an analogy can be made with the classical laminate theory (CLT) used for continuous fibre composites, with each printed filament considered as a continuous fibre.

The CLT makes it possible to write the behaviour law of a balanced and symmetric laminate in a plane stress state as (Equation (7)) [43]:

(7) $$\left(\begin{array}{c} N_{x}\\ N_{y}\\ N_{xy}\\ M_{x}\\ M_{y}\\ M_{xy} \end{array}\right)=\left(\begin{array}{cccccc} A_{11} & A_{12} & 0 & 0 & 0 & 0\\ A_{21} & A_{22} & 0 & 0 & 0 & 0\\ 0 & 0 & A_{33} & 0 & 0 & 0\\ 0 & 0 & 0 & D_{44} & D_{45} & D_{46}\\ 0 & 0 & 0 & D_{54} & D_{55} & D_{56}\\ 0 & 0 & 0 & D_{64} & D_{65} & D_{66} \end{array}\right)\times \left(\begin{array}{c} \varepsilon_{xx}\\ \varepsilon_{yy}\\ \gamma_{xy}\\ k_{x}\\ k_{y}\\ k_{xy} \end{array}\right)$$

where *N_i_* is the resultant force and *M_i_* the resultant moment applied to the laminate in the *i* direction; $\varepsilon_{xx}$ and $\varepsilon_{yy}$ are the longitudinal and transverse strains, respectively, $\gamma_{xy}$ the distortion, *k_x_* and *k_y_* the bending curvatures, and *k_xy_* the torsion; and *D_ij_* are the bending stiffness coefficients. The coefficients of the extensional stiffness (*A_ij_*) are determined thanks to Equation (8):

(8) $$A_{ij}=\sum_{k=1st\;ply}^{nb\;of\;plies}Q_{ij}^{k}\times e_{k},$$

where $Q_{ij}^{k}$ is the coefficient of the stiffness matrix of an orthotropic unidirectional ply *k*, and *e_k_* is its thickness. Considering *l* the direction of the filaments, and *t* the direction perpendicular to *l* in the plane of the ply, the compliance matrix corresponding to the stiffness matrix can then be written (Equation (9)):

(9) $$\left(\begin{array}{c} \varepsilon_{l}\\ \varepsilon_{t}\\ \gamma_{lt} \end{array}\right)=\left(\begin{array}{ccc} \frac{1}{E_{l}} & -\frac{\nu_{tl}}{E_{t}} & 0\\ -\frac{\nu_{lt}}{E_{l}} & \frac{1}{E_{t}} & 0\\ 0 & 0 & \frac{1}{G_{lt}} \end{array}\right)\times \left(\begin{array}{c} \sigma_{l}\\ \sigma_{t}\\ \tau_{lt} \end{array}\right).$$

where *E_i_*, $\varepsilon_{i}$, and $\sigma_{i}$ are the Young’s modulus, strain, and stress along the *l* or *t* direction, respectively, $\gamma_{lt}$ is the distortion, $\tau_{lt}$ the shear stress, *G_lt_* the shear modulus and *v_lt_*, and *v_tl_* are the Poisson’s ratios. To apply the CLT to our material, four independent parameters are therefore needed: *E_l_*, *E_t_*, *v_lt_*, and *G_lt_*. The previous measurements on unidirectional samples make it possible to deduce these parameters. The 0° samples give us the Young’s modulus along the direction *l* (Equation (10)) and the Poisson’s ratio *v_lt_* (Equation (11)):

(10) $$E_{l}=E_{x}^{0};$$

(11) $$\nu_{lt}=\nu_{xy}^{0}.$$

The 90° samples give us the Young’s modulus along the direction *t* (Equation (12)):

(12) $$E_{t}=E_{x}^{90}.$$

Finally, *G_lt_* can be calculated with the measurements of the ±45° samples, as the slope of the linear part of the curve following the formula (Equation (13)):

(13) $$\frac{1}{2}\sigma_{xx}^{\pm 45}=G_{lt}\left(\varepsilon_{xx}^{\pm 45}-\varepsilon_{yy}^{\pm 45}\right).$$

All the results are summarised in Table 2. The formula $\frac{\nu_{lt}}{E_{l}}=\frac{\nu_{tl}}{E_{t}}$ is also verified, with $\nu_{tl}=\nu_{xy}^{90{}^{\circ}}$.

In a second step, applying the CLT, the theoretical Young’s modulus of the QI sample can be calculated, as well as its Poisson’s ratio (Table 3). The values are compared to the experiment, and a difference of only 1.2% and 5.4% for the Young’s modulus and the Poisson’s ratio, respectively, is noticed. It shows that the classical laminate theory can be applied to 3D-printed CB/PLA samples and demonstrates that the filaments deposited by the printer can be considered continuous fibres despite the porosities inherent to the printing process.

### 3.2. Repeated Progressive Tensile Loading

**Figure 6:**
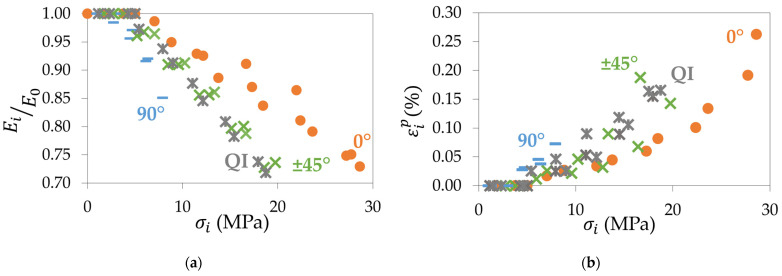
For the four configurations: (**a**) evolution of the ratio between the secant moduli and the Young’s modulus with the maximum stress of the corresponding tensile cycle; (**b**) evolution of the permanent strain with the maximum stress of the corresponding cycle.

Following the establishment of the mechanical properties of the 3D-printed CB/PLA, a series of repeated progressive tensile loadings was performed to better understand the evolution of the mechanical properties during a tensile test. As explained in Section 2.2, the secant modulus and the permanent strain were measured up to 80% of the elongation at break. Figure 6a shows the evolution of the secant moduli compared to the Young’s modulus for all the samples versus the maximum stress of each corresponding loading-unloading cycle. This graph highlights the kinetics of loss of stiffness during tensile testing: the 90° samples see their secant moduli decrease faster than the ±45° samples and the 0° samples. The QI samples have a similar tendency as the ±45° samples, intermediate between the 90° and 0° samples. However, the ratio between the initial and final stiffness is 85% for 90° samples, whereas it drops to almost 70% for the other specimens. Thus, the 90° samples lose about 15% of their initial stiffness, while the other configurations lose almost 30%. The evolution of the permanent strain is shown in Figure 6b. It can be seen that for a given stress, the permanent strain is higher for the 90° samples. It confirms the observations made for the secant moduli: the 90° raster angle tends to see its mechanical properties deteriorate faster than the other specimens. At higher stresses, the same hierarchy as for the secant moduli remains: the 0° samples degrade at a slower rate than the ±45° and QI specimens that present a similar behaviour. The 0° samples, however, reach higher permanent strains because they are undergoing higher stresses than the other configurations. Once again, the 0° raster angle demonstrates superior mechanical characteristics compared to the other raster angles.

### 3.3. Damage Monitoring by Acoustic Emission

To better understand the degradation of the tensile mechanical properties, damage monitoring was conducted by acoustic emission. The sound wave propagation was measured in the first step for all configurations (Table 4). The sound waves propagate faster in the 0° and QI samples than in the ±45° and 90° samples, due to differences in the microstructure. In a 0° sample, a sound wave just has to follow the filament from one point to another within the material. In a 90° sample, it has to circulate from one filament to another, and the inherent porosity between two printed filaments slows down its progression. The ±45° lay-up is intermediate between these two structures. Finally, the QI samples contain 0° layers, which explains the similarity of their sound wave propagation speed, within the standard deviation, with the 0° samples.

**Figure 7:**
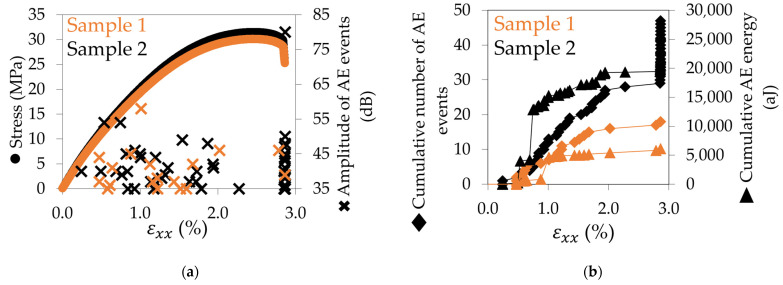
AE results for two 0° samples: (**a**) amplitude of AE events against stress–strain curve and (**b**) cumulative number of events and cumulative AE energy versus longitudinal strain.

The acoustic emission occurring within 0° samples was first measured. The amplitude of the AE events, the cumulative AE energy, and the cumulative number of AE events are presented for two 0° specimens in Figure 7. The results obtained show the reproducibility of the experiment between sample 1 (in orange) and sample 2 (in black). In Figure 7a, it can be seen that AE events are heard as soon as the end of the elastic domain of the sample. The amplitude (cross points) is included between 35 dB and 80 dB. Nothing is heard after the maximum stress is reached and before the failure. The same conclusions can be deduced from Figure 7b with the cumulative number of AE events (diamond points). In addition, Figure 7b shows that, for the two samples, the cumulative AE energy (triangle points) follows the same trend as the cumulative number of AE events up to the failure.

**Figure 8:**
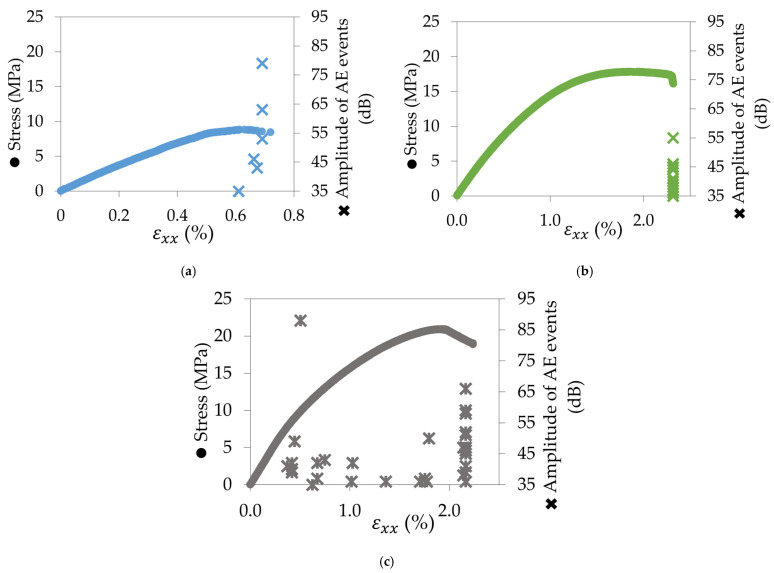
Amplitude of AE events against stress–strain curve for (**a**) 90°, (**b**) ±45°, and (**c**) QI samples.

For the other configurations, the reproducibility of AE recording was also verified. Figure 8 shows the results for one specimen of each configuration. In contrast to the 0° samples, no AE events are heard before the failure in the 90° raster angle (Figure 8a) and the ±45° raster angle (Figure 8b). The amplitude of the events is included between 35 dB and 80 dB for the first one and 35 dB and 55 dB for the second one. However, for the QI sample (Figure 8c), the same behaviour is observed as for the 0° sample: AE events are heard between the end of the elastic domain and the maximum stress and at the failure. It can be explained by the presence of 0° layers in the sample.

### 3.4. Fractography

**Figure 9:**
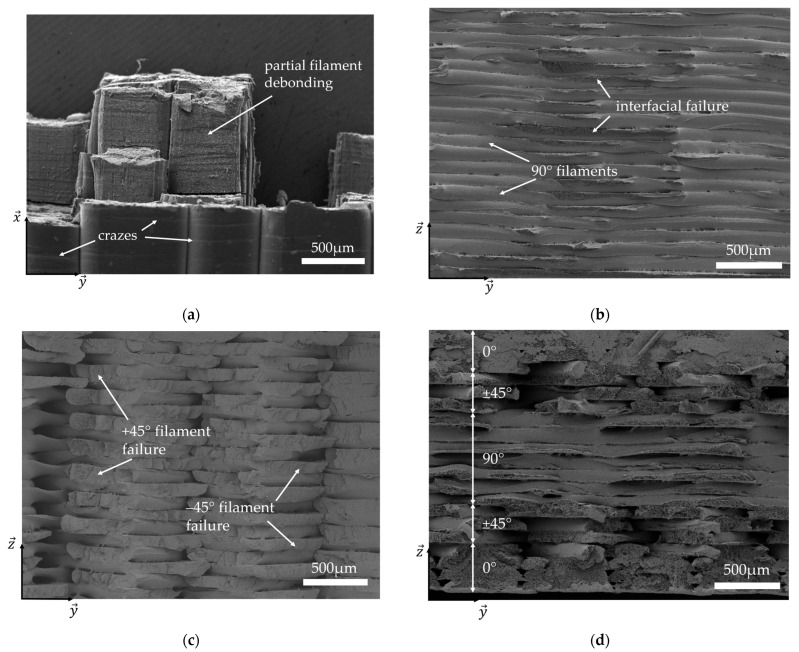
Fracture surfaces observed by SEM of (**a**) 0°, (**b**) 90°, (**c**) ±45°, and (**d**) QI samples.

To better explain the damage monitoring by acoustic emission, the fracture surface of the specimens was observed by SEM. The failure of the specimens at 0° is due to the breaking of filaments, but not all in the same plane, resulting in partial debonding from one filament to another between two superimposed layers (Figure 9a). On the 90° fracture surface (Figure 9b), it can be seen that the failure is due to the debonding of the adjacent filaments within the same layer. It occurs along the same cross-section of the sample and correlates with the results in Section 3.1: the interface between the filaments is the weak point of the 90° sample; the filaments tend to separate from each other rather than enduring the increase in stress that is applied to the interface. For the ±45° fracture surface (Figure 9c), the breaking of filaments perpendicular to each other can be observed. The failure of the 0° and ±45° samples is clearly due to the failure of the filaments, as opposed to the interfacial failure in the 90° sample. These observations explain the difference in mechanical properties in Section 3.1 as the filaments can undergo higher stresses than the interfaces between them. Indeed, the interface between two filaments is created by depositing a hot filament next to a cooling one, which reheats the borders of the previously deposited filament to weld it to the new one but makes this interface weak. On the QI fracture surface (Figure 9d), the combination of all the different layer failures can be observed, with the same failure mechanisms as those observed in the other specimens.

**Figure 10:**
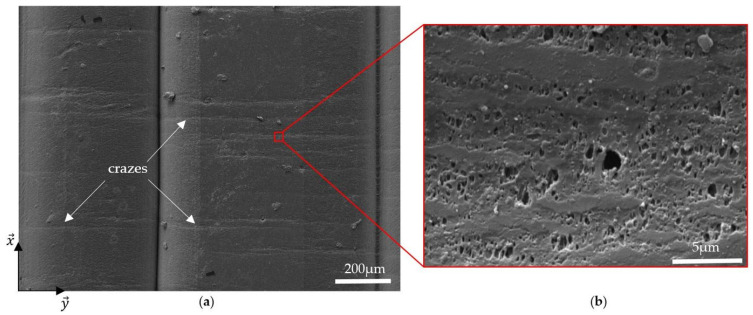
SEM images of the surface of a 0° sample after failure (**a**) and zoom in on crazes (**b**).

When looking closer to the 0° sample (Figure 9a), white lines perpendicular to the filaments can be seen at the surface of the sample at the bottom of the SEM image. In Figure 10a, these lines can be better observed. The working distance in the SEM was changed from 16.27 mm to 9.10 mm to zoom in on them (Figure 10b). It can be seen that horizontal bits of material are linked by vertical polymeric chains that result from the ductile deformation of the material, creating clouds of holes. This type of damage, known as crazing, has already been observed in the literature on conventional polymers [44] as well as on 3D-printed samples by FDM [45]. Conway and Pataky [46] studied the development of crazes in compression-moulded and 3D-printed FFF ABS specimens. They explained that craze walls are created perpendicular to the loading direction while amorphous polymer chains are realigned along this direction, connecting the walls together. These chains become load-bearing, and once they break, the stress is redistributed between them. As the local stress increases elsewhere in the specimen, crazes are created there, which results in a repartition of bunches of crazes all along the sample. Eventually, too many chains fail in the same heavily crazed area, leading to cracks and failure.

**Figure 11:**
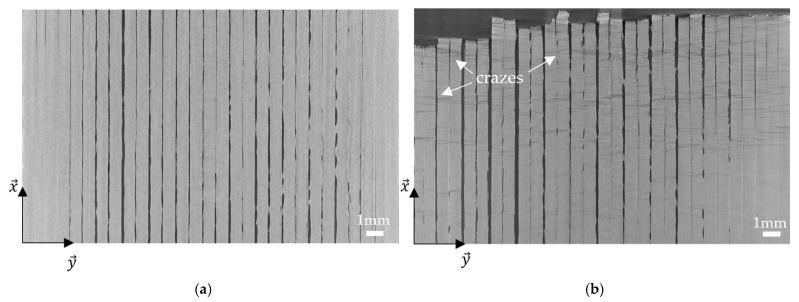
Micro-CT images of the core of a 0° sample before (**a**) and after (**b**) monotonic tensile test.

To better understand the evolution of the crazes in the samples, micro-CT scans of the four kinds of specimens were performed at three different steps: initial, i.e., when the sample just got printed; at 80% of the elongation at break; and after failure. An example of a 0° sample at the initial step and after failure is presented in Figure 11. Before the tensile test, no damage was observed to the sample but to the printing paths of the filaments. It is true for the 90°, ±45°, and QI specimens as well. However, after failure, crazes are observed on the 0° sample and the 0° layers of the QI sample, not only on the surface but also in all the 0° layers of the samples. Therefore, the images at 80% of the elongation at break were analysed, and crazes were also noticed, but less numerous. These observations were made for the 0° sample as well as for the QI sample. It means that, at least at 80% of elongation at break, a few crazes are already developed. It can be supposed that these crazes are developed all along the tensile test, but their initial state is smaller than the voxel resolution of 12 μm used here, so they cannot be seen. Since these crazes were not observed in the 90° and ±45° specimens, it can be supposed that the creation of the crazes is the source of the acoustic emission activity heard for the 0° and QI samples and analysed in Section 3.3. No crazes were developed in the 90° and ±45° samples, which means no AE events occurred before their failure. To finish, no other damage mechanisms were observed on the samples tested at 80% of the elongation at break, which suggests that when visible damage occurs, apart from the crazes, it directly leads to the failure of the samples.

**Figure 12:**
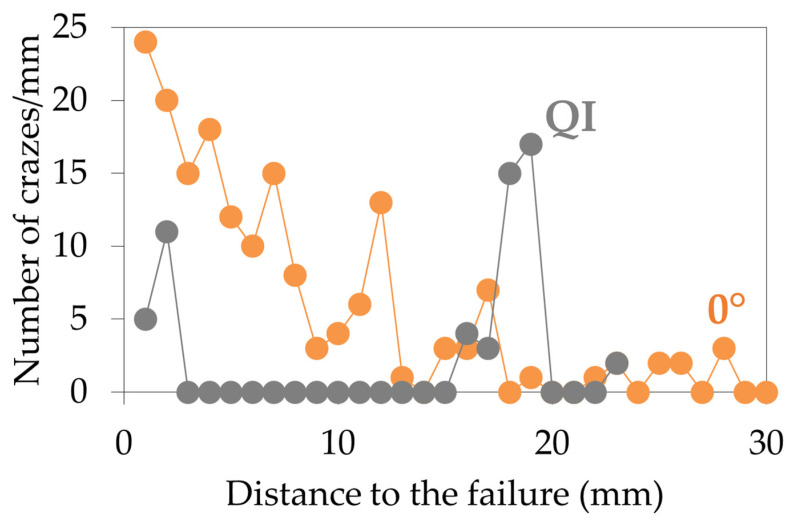
Number of crazes by millimetre along a central filament versus the distance to the failure for a 0° sample and a QI sample after tensile testing.

The crazes per millimetre were counted along a central filament taken on an image at the core of a 0° sample and on an inner 0° layer of a QI sample after failure (Figure 12). It can be seen that the crazes are less and less numerous as the failure gets farther for a 0° sample. They have an overall tendency to be more numerous close to the failure, but the crazes are also organised by bunches: there are zones where they are more numerous, like in the fourth, seventh, twelfth, and seventeenth millimetres. This correlates with the definition of crazes: several bunches of crazes were created before one heavily crazed area failed. As opposed to this, the QI sample does not especially have more crazes close to the failure. A peak is even observed in the eighteenth and nineteenth millimetres, where they are more numerous than next to the failure. This difference in behaviour can be explained by the presence of 90° and ±45° layers in the QI sample.

### 3.5. Evolution of the Electrical Resistivity

Before the tensile tests, the initial electrical resistance of the samples was measured, and the initial resistivity was also calculated according to Equation (1). The results are summarised in Table 5 for the four configurations. As already noticed in a previous study [22], the electrical resistance of the 90° sample is higher than that of the 0° sample. This is due to the fact that electricity can propagate easily along a printed filament in a 0° sample. Conversely, in a 90° sample, the electricity encounters obstacles such as the interfaces between two adjacent filaments, which slows down the progression of the current. The initial resistance of the ±45° samples is intermediate between that of the 0° and 90° samples because the path of electricity is easier through the filaments at 45° than at 90°, but it still has to cope with the interfaces between the filaments. Finally, the electrical resistance of the QI samples is logically slightly higher than that of the 0° samples since they contain different raster angles.

**Figure 13:**
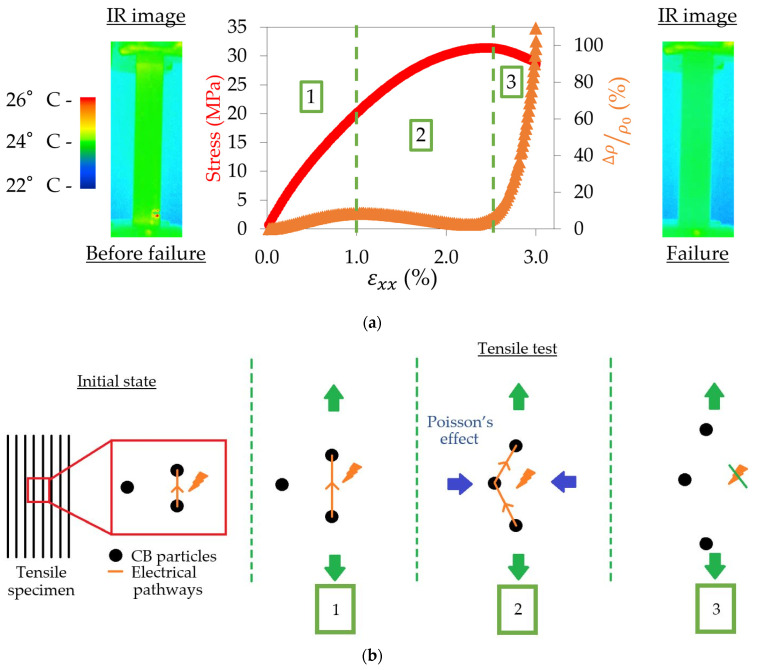
(**a**) Stress–strain curve in comparison with the evolution of the relative variation of resistivity for a 0° sample and IR images of the tensile specimen. (**b**) Illustration of the consequence of the evolution of the microstructure of the CB/PLA on its resistivity depending on the tensile test stage of the 0° sample.

Following the protocol described in Section 2.2 and Figure 3a, the electrical resistance was recorded all along the tensile tests, and the resistivity was then calculated thanks to Equation (6) and the measurement of the longitudinal and transverse strains. Figure 13a shows the evolution of the relative variation of resistivity for a 0° sample, defined by Equation (14):

(14) $$\frac{∆\rho }{\rho_{0}}=\frac{\rho_{0}-\rho (t)}{\rho_{0}}$$

where $\rho_{0}$ is the initial resistivity of the sample and ρ(t) is its resistivity at time *t*. First, the surface of the sample was studied by an infrared camera throughout the tensile test. Two images, before and after failure, are presented in Figure 13a.

It can be seen in Figure 13a that the sample is at room temperature all along the test and that the temperature slightly increases at the end of the test around the failure zone of less than 4 °C. Therefore, it demonstrates that the evolution of the electrical resistance is not due to the potential heating of the sample during the tensile test. Then, the evolution of the resistivity is analysed. Three different stages can be observed: an increase in resistivity when the sample is in its elastic domain; a decrease in resistivity between the beginning of the plastic domain and the reaching of the maximum stress; and finally, a huge increase in resistivity up to the failure of the sample. This behaviour has already been observed, and a hypothesis of explanation was suggested by Tirado-Garcia et al. [24]. An illustration of the mechanism is proposed in Figure 13b. In the initial state of the sample, electrical pathways are created between the carbon black particles. These paths can be within a filament or between two adjacent filaments, but as said before, the interface between the filaments makes it harder for the electricity to propagate. Therefore, the electricity always chooses the easiest path and most likely mainly propagates within a filament when possible. In the first stage of the tensile test, the distance between the particles increases as the sample has an elastic behaviour and sees its length increase. It results in a slow increase in resistivity as the paths between some particles might be suppressed due to the distance between them being too high, but the majority of the pathways are maintained. Once the sample enters its plastic behaviour in stage two, the particles are brought together in the vertical dimension due to the Poisson effect influence. It is traduced by a reduction of the transverse dimension while the longitudinal dimension still increases. This phenomenon creates new paths for the electrons within a filament as the interparticle distance decreases, leading to an increase in the conductivity overall. This trend reverses in stage three once the maximum stress is reached, as the interparticle distance is too high and the development of crazes prevents the electricity from circulating, leading to the progressive destruction of electrical pathways, thus causing a huge increase in resistivity up to the failure of the sample.

**Figure 14:**
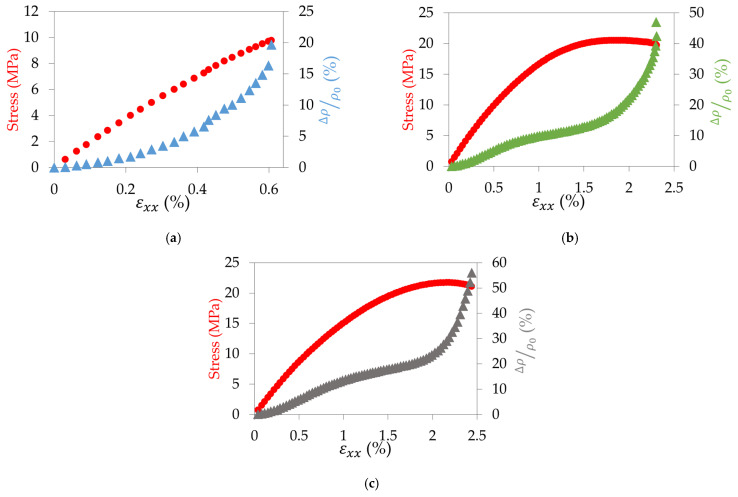
Stress–strain curve in comparison with the evolution of the relative variation of resistivity for (**a**) a 90° sample, (**b**) a ±45° sample, and (**c**) a QI sample.

This study was also conducted for the other configurations to highlight the different inter- and intra-filament electrical behaviours. In Figure 14a, the evolution of the resistivity of a 90° sample is presented versus its stress–strain curve. It can be seen that the resistivity increases exponentially up to the failure. This phenomenon can be explained by the fact that contrary to the 0° samples, the electrical pathways are governed by the progressive enlargement of the inter-filament gaps as the electrons have to propagate from one filament to another. The sporadic connections between the filaments locally fail one after the other more and more quickly with the increase in stress, leading to a high increase in the resistivity. The Poisson’s ratio of the 90° samples is too small to compete with the destruction of the electrical pathways (Table 1). For the ±45° sample (Figure 14b), both inter (0°-like) and intra (90°-like) filament electrical behaviours are contributing to the increase in resistivity. Indeed, the orientation of the filaments, which does not allow the electric current to flow along the entire length of the sample in a single filament as for the 0° sample, forces it to circulate from one filament to another. This leads to an overall increase in resistivity, slowed down by the Poisson effect in stage two. For the QI sample (Figure 14c), the behaviour is similar to that of the ±45° sample, as the same phenomenon explained before occurs, and a balance between the different layers is made.

**Figure 15:**
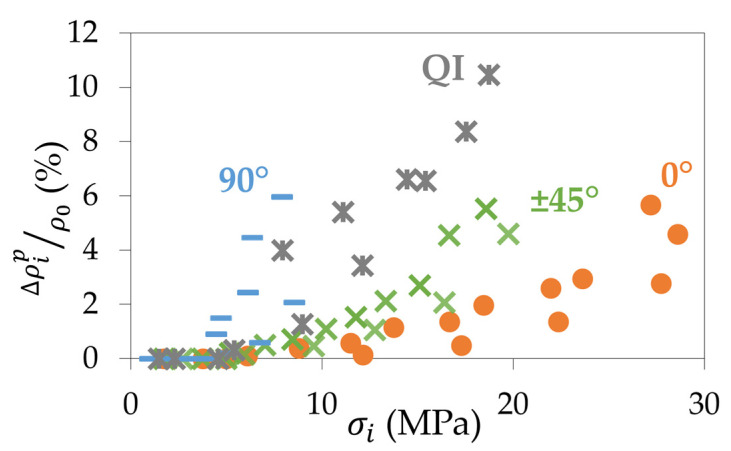
Evolution, for the four configurations, of the relative variation of resistivity after each unloading during the repeated progressive tensile tests as a function of the maximum stress of the corresponding loading-unloading cycle.

The electrical resistance was also recorded throughout the repeated progressive tensile tests. The relative variation of resistivity ∆ρipρ0 was determined thanks to Equations (6) and (14) from the permanent electrical resistance $R_{i}^{p}$ measured after each unloading (as defined in Figure 4). Figure 15 shows the results obtained for the four configurations. It can be seen that the resistivity of the 90° samples increases faster than for a QI, a ±45°, and a 0° sample. The relative variation of resistivity reaches about 6% for the 90° samples, as well as for the ±45° and 0° samples. However, for the latter two, the increase is much slower and the initial resistance is much lower (Table 5), resulting in better electrical conductivity in the end. The QI sample shows an intermediate behaviour between the 90° and the ±45° samples but reaches an increase in its relative variation of resistivity of up to 11% (Figure 15). In terms of mechanical and electrical coupling, the kinetics of the curves in this graph correlate well with the evolution of the mechanical properties in Figure 6. These results demonstrate that the unidirectional 0° samples exhibit both superior mechanical and electrical properties. Indeed, the 0° samples not only have the highest initial values for stiffness and conductivity but also the slowest decrease in these values during tensile loading.

Figure 15 shows that, whatever the type of CB/PLA samples, the electrical resistance increases during repeated progressive tensile tests. It means that if a potential 4D-printed structure is used several times and heated by the Joule effect, increasing the voltage would be needed throughout the experiment to achieve similar temperatures, which is to be taken into account in the design of a future actuator.

## 4. Conclusions

In this paper, an original electro-mechanical study of 3D-printed CB/PLA samples was presented. First, a mechanical characterisation was conducted on different configurations of 3D-printed rectangular samples: 0°, 90°, and ±45° samples, as well as a quasi-isotropic sample: [0_6_/±45_5_/90_5_]_S_. Results showed a high anisotropy of the material, leading to the hypothesis of considering it as a continuous fibre composite to apply the classical laminate theory. This analogy was demonstrated for these 3D-printed CB/PLA specimens. Then, repeated progressive tensile tests were performed in order to investigate the evolution of the properties of the material during tensile loading. It was shown that the 0° samples exhibited a slower decrease in secant moduli and a slower increase in permanent strain compared to the ±45° and 90° samples, while the QI samples demonstrated a similar trend as the ±45° samples. Acoustic emission monitoring was carried out and revealed no acoustic activity before failure in the 90° and ±45° samples, whereas the 0° and QI samples showed a noticeable amount of AE events throughout the test. To identify the failure mechanisms, the fracture surfaces of the samples were studied by SEM. On the 0° layers, crazes were identified, and a micro-CT analysis was performed at three stages: initial, at 80% of the elongation at break, and after failure. This way, the creation and evolution of crazes were demonstrated in the 0° layers of 0° and QI samples, explaining their acoustic activity. Finally, the electrical resistance of the four types of samples was measured all along the tensile tests and after each unloading of the repeated progressive tensile tests. The 0° samples exhibited a unique evolution of their resistivity during tensile loading in three distinct phases (increase/decrease/increase), the second phase being explained by the Poisson effect. For all the kinds of samples, it was also shown that the resistivity ratio after unloading increased during the repeated progressive tensile tests. Consequently, in the case of the repeated use of an electro-thermally triggered 4D-printed actuator, the voltage would need to be increased to compensate for the higher resistance to still achieve the same temperature within the structure. However, results show that this increase will be less significant when using a unidirectional 0° sample. Indeed, the 0° samples have the best initial mechanical and electrical properties and are the least affected by repeated tensile loading, showcasing slower changes in secant moduli, permanent strain, and resistivity, thus demonstrating their superior mechanical and electrical performance compared to their 90° and ±45° counterparts. These findings extend to electro-thermally triggered 4D-printed actuators, making unidirectional longitudinal printing patterns more suitable for such applications. In conclusion, this study paves the way to a better understanding of the electro-mechanical coupling for the design of a 4D-printed conductive and thermally triggered actuator.

## Figures and Tables

**Table 1:** Mechanical properties.

Raster Angle	Young’s Modulus (MPa)	Poisson’s Ratio	Maximum Stress (MPa)	Failure Stress (MPa)	Elongation at Break (%)
0°	2542 ± 15	0.42 ± 0.03	29.6 ± 1.7	26.8 ± 2.0	3.47 ± 0.39
±45°	2108 ± 86	0.40 ± 0.03	19.6 ± 2.3	18.7 ± 2.5	2.36 ± 0.15
90°	1744 ± 108	0.25 ± 0.01	8.3 ± 0.9	7.8 ± 1.2	0.70 ± 0.07
QI	2187 ± 125	0.37 ± 0.05	21.8 ± 0.8	21.7 ±0.7	1.89 ± 0.13

**Table 2:** Independent parameters of the orthotropic law for 3D-printed CB/PLA.

*E_l_* (MPa)	*E_t_* (MPa)	*v_lt_*	*G_lt_* (MPa)
2542	1744	0.42	761

**Table 3:** Comparison between the CLT and the measured values for a QI 3D-printed CB/PLA sample.

Sample	Parameter	CLT Value	Measured Value	Percentage of Difference
QI	*E_x_* (MPa)	2160	2187 ± 125	1.2%
	*v_xy_*	0.35	0.37 ± 0.05	5.4%

**Table 4:** Sound wave propagation speed in the different types of specimens.

	0°	90°	±45°	QI
Sound wave propagation speed (m/s)	2560 ± 157	1620 ± 115	1900 ± 100	2760 ± 81

**Table 5:** Initial electrical resistance and resistivity of the different 3D-printed CB/PLA specimens.

	0°	90°	±45°	QI
*R*_0_ (ohm)	355 ± 11	683 ± 15	457 ± 12	389 ± 32
$\rho_{0}$ (ohm.mm)	119 ± 2	228 ± 6	155 ± 2	134 ± 12

## Data Availability

Data are contained within the article.
